# Leveraging artificial intelligence in bioacoustics for animal health monitoring and early diagnosis in veterinary medicine

**DOI:** 10.3389/fvets.2026.1816544

**Published:** 2026-04-24

**Authors:** Hannah Rideout, Anthony D. Whetton

**Affiliations:** 1 Veterinary Health Innovation Engine, School of Veterinary Medicine, Faculty of Health and Medical Sciences, University of Surrey, Guildford, United Kingdom; 2 School of Biosciences, Faculty of Health and Medical Sciences, University of Surrey, Guildford, United Kingdom

**Keywords:** acoustic listening, acoustic sequences, acoustic sound, animal health, animal sounds, anthropogenic sounds, bioacoustic analysis, veterinary medicine

## Abstract

The study of animal communications, termed zoosemiotics, includes the sub-field of bioacoustics, the study of the production, transmission, and reception of animal sounds. It is becoming increasingly apparent that inter- and intra-species communication is sophisticated with sound playing a major role in this signaling. Artificial intelligence-led research can be employed to understand and combine recorded multi-level data (sound, vision, odors) to classify animal health and identify interventions, also determining critical time-points for intervention. This can include subgroup discovery and trajectory analysis as essential elements in developing animal specific identification of failure to thrive or ill health. It is important that animals, carers, and veterinarians receive as early a diagnosis as possible to predict trajectory and plan care needs and interventions. However, the use of quantitative data for evidence-led interventions based on sound have not yet been developed. Here we look at advances in bioacoustics and provide a framework to determine where early diagnosis and animal health improvements can be made via understanding of behavior and oral sound production.

## Introduction

There has been a burgeoning interest in communication within and between species. This, in part, is based on technological advances in artificial intelligence and machine learning, leading to major new insights (1).

The inception of the academic approach to the field came with The Question of Animal Awareness (2), a formative contribution to our thinking on communication with other species. The book poses essential questions relevant to ethics, anthropology, consciousness, and two-way communication between species. As an example of a more trivial advancement in computational tools, a word search on this book shows the term “bats” used 40 times, “primate” 16 times, “chimpanzee” 108 times and “great ape” 11 times. “Illness,” “disease,” and “veterinary” are words never used in the book and “medicine” is used only once with important context: “Animal surrogates have been invaluable in the analysis and explanation of many biological phenomena, including some aspects of behavior, such as learning. The resulting knowledge and understanding have had many important applications to human medicine in particular and to human affairs in general.” In other words, non-human species have served us well in developing an understanding of disease. Our understanding of mouse gene function and its relation to behavior is vast. In part this has been achieved with ultrasonic vocalization and video surveillance of behaviors in transgenic or gene knockout mice (3).

Understanding intra-species communication, where video and sound recordings (including those beyond the human hearing range) meet artificial intelligence approaches and observations on societal organization and behavior, provides an exciting area of research. However, some key questions we must consider are: how can it benefit animal welfare in respect of livestock and companion animals? and is there potential for exploitative behavior by humans based on furthering our understanding of non-human animal communication?

Here we consider aspects of research on non-human communication from the perspective of developing a knowledge base for improving veterinary care, animal health and precision veterinary medicine.

## Bioacoustics and ecoacoustics

The study of animal communications, termed zoosemiotics (4) includes bioacoustics, which is the study of the production, transmission, and reception of animal sounds (5), whereas ecoacoustics studies broader ecological processes and environments (6). Bioacoustic techniques can be used for species detection, location, population monitoring, and verification of how human activities affect wild animal behavior (7), with methods being increasingly automated (e.g., remote multi-site acoustic data acquisition across frequencies). Bioacoustic research is a valuable data source for monitoring and understanding animal behavior (8). For a thorough review of this area see Bakker (9). Soundscapes encompass a mixture of biotic, abiotic, and anthropogenic sounds; all important to understand or monitor both environmental and ecosystem changes (10). Bioacoustics research within animal science generally focusses on avoidance/protection of negative experiences, pertaining to the five freedoms: from hunger and thirst, from discomfort, from pain, injury, or disease, expression of normal behavior, from fear and distress (see Table 1) (5). This is determined using scoring methods or physiological assessments, both invasive and non-invasive (11). However, research suggests there has been a rise in the significance of including positive experiences as measures of welfare (12, 13). There is a view that we can move beyond enabling the five freedoms to providing a structure for a life worth living in non-humans (12). Thus, vocalizations have been used to classify between positive and negative sounds in species such as goats, chickens, and horses (14, 15). Such sounds offer opportunity in terms of predicting trajectory and diagnosis in disease. In a veterinary context, bioacoustics provides a means of objectively capturing and analyzing sounds and vocalizations, already critical to clinical assessments, e.g., respiratory sounds and gut motility.

**Table 1:** The five freedoms and how bioacoustic research can aid their implementation.

Freedoms (Freedom from)	Bioacoustic benefit
Hunger and thirst	Studying behaviors and sounds to show preferences to feed and ensure each animal has appropriate access to feeders and drinkers.
Discomfort	Analysis of behaviors via video footage can highlight discomfort.
Pain, injury and disease	Sounds and behavioral signs can indicate spread of disease and highlight pain indicators in many species.
Fear and distress	Sound can be analyzed to understand distress calls in prey species.
Freedom to express normal behaviors	Analysis of behaviors using sounds can indicate ‘happiness’ in many species and video footage can analyze for stereotypical behaviors associated with stress.

Bioacoustics has been demonstrated as a successful monitoring tool at low cost, meaning a reduced outlay for meaningful research, used across a wide range of species. Often technologies contain built-in analytical tools for better analysis, as well as offering a data collection method with minimal human interference (16). Speech recognition software is an example of bioacoustics application-to-audio analysis focussing on classification of scene vs. event (e.g., the rainforest vs. a passing species). We address this in more detail below.

## Analytical methods employed in bioacoustics

There are three main analytic approaches for identifying and utilizing sound signals (17). The first is based on classification, description, and analysis of vocalizations in various contexts within a single species, allowing connections between sounds and behaviors. The second is a combination of sounds with behavioral context to understand how the sounds may affect behavior and emotions of the receiver, and vice versa. The third is how sound production mechanisms are involved in the processes of vocalizing. A full account of such methods, techniques and software available for bioacoustics monitoring are available in a review by (18).

Bioacoustics is a field combining interdisciplinary sciences of artificial intelligence, biology and acoustics, using technologies to record and analyze large data sets (19). Sound signals are species-specific data sets that can be interpreted and perceived by both animals and humans. Neurophysiological and anatomic knowledge of sound emission and reception are paramount to this field of interest as, depending on the type and method of processing signals, behavioral assessments can be achieved from signal exchange (18). For example, work on bat communications and language contextualized to and inclusive of hearing loss in humans, gives clarity on methods and approaches pertinent to understanding livestock communications (20).

Sound waves travel at temperature-dependent speed through media such as air and water, and at a range of frequencies. Audible frequencies for humans range from 20 Hz to 20 kHz, while in the animal kingdom, ranges tend to fall above or below this, existing as a world of sound only accessible to us via technology. As an example, mice have a range from 1 kHz up to approximately 100 kHz.

Signals below this range are termed infrasound; they travel far and wide, being generated and understood as communications by species such as whales and elephants. Alternatively, signals above this range are ultrasound, more commonly known as echolocation in bats and dolphins (18). As many animals produce sounds outside of the standard human hearing range, there is a need for the use of acoustic listening and AI tools to analyze and interpret these communications. Many experiments on mice for human genetics purposes have used such sophisticated listening. This includes sophisticated modeling such as a Markov model of behavioral or acoustic sequences, used to analyze, segment, and predict sequences of ultrasonic vocalizations (USVs) in social interactions, This is not to say that an experienced stockperson could not qualitatively tell something of the state of a flock/herd via the sounds that the animals produce, but there is presently no science base to this, and it does not include the inaudible.

“Dip” listening allows for the ability to catch brief glimpses of acoustic sound with decreased background noise levels (21). Exploitations of these dips can allow animals to pick out sounds in overwhelming environments for intraspecies communication such as treefrogs selecting a single mate through a cacophony of breeding choruses. However, struggles with temporal pattern recognition may reduce the benefits, which in turn could negatively impact the evolution of male signaling strategies in louder environments (21). With respect to livestock, acoustic listening in poultry sheds is technically easy, but dip listening is far more of a challenge. Neethirajan et al. (22) found that the vocal responses of poultry to visual and auditory stressors are extremely contrasting, indicating that hens can use vocal patterns to adaptively respond to environmental changes. Additionally, their research found that induction to stress leads to immediate alterations in vocal behaviors of hens. Therefore, acoustic listening can be applied to analyze stress vocalizations for the early detection of distress in poultry sheds. But poultry cannot learn vocalization, as seen in songbirds.

Passive acoustic monitoring overtook traditional survey methods in the 2010s as it was found to be more effective (23). Unfortunately, much of the available wildlife monitoring equipment is expensive, heavy, and labor intensive/difficult to install in certain areas. Therefore, smartphone recorders and other inexpensive recorders (e.g., AudioMoth)^1^ are being developed and tested to negate these difficulties, making passive monitoring more widely used. Within the agricultural sector, it is generally recognized that any increases to running costs must generate an improvement in yield to warrant uptake.

### Technologies

#### Microphones

**Figure 1:**
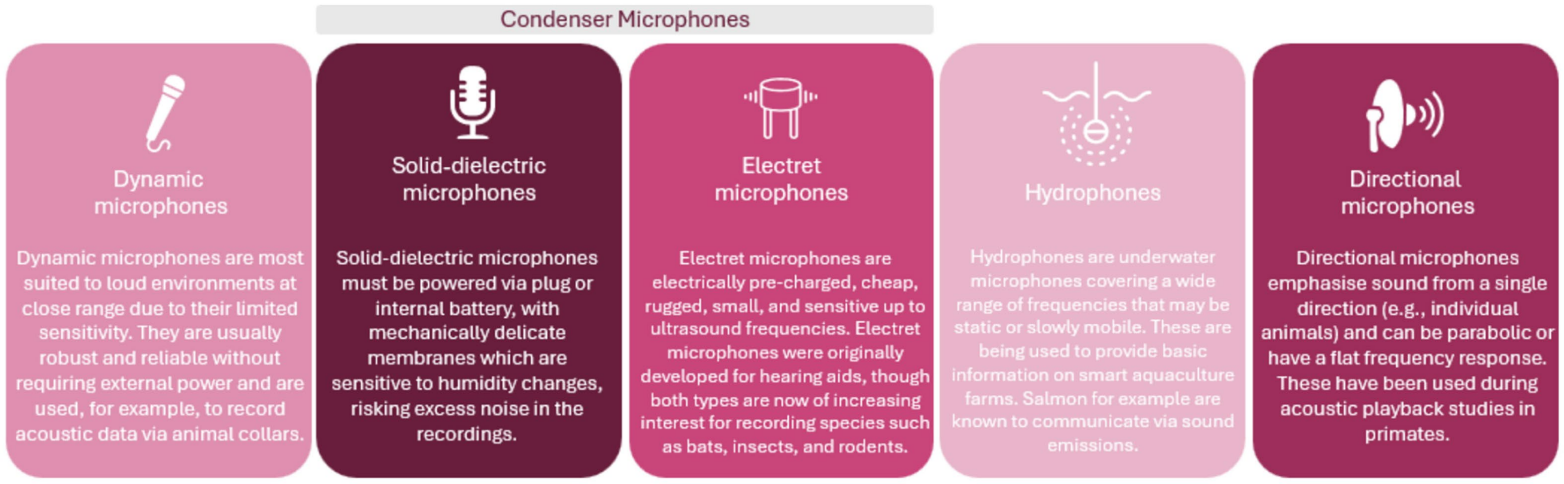
Types of microphones available for use for bioacoustic research (141–145).

Various types of microphones are now available on the market to use within bioacoustics research. Each have their advantages and disadvantages making them suitable to unique circumstances. The types of microphones and their uses are outlined in Figure 1.

### Digital recording

Automatic Recording Systems (ARS) reduce person-hours in the field and connect to a computer for scheduling (24). Energy requirements and storage capacity remain limiting factors. Rapid expansion of data sets is associated with digital recording, though with emerging technologies, data can be analyzed effectively at speed.

Bioacoustic sounds are often classified for analysis. However, significant variation, due to factors including age, sex, location, and date, makes it difficult to define such categories. Furthermore, factors including artifact, sound propagation effects, angular aspects of sound emission, geographic variation, and changes in repertoire over time also add to recording difficulty (25).

### Video recording

As technologies have improved, powerful multimedia techniques have been established related to bioacoustics analysis. The inclusion of visual and audio-visual (AV) recording enables segmentation, motion-based indexing, pattern indexing, and flexible content descriptions (26). Video recording is a non-invasive way to provide more context to acoustic data alone, and therefore, more information about communication methods. In some cases, video recordings can also offer extra insights into behavioral studies that may be overlooked by audio recordings alone (e.g., keeper activity in domestic animals, failure to thrive in pigs) (27). Video monitoring is advantageous as cameras can be placed in multiple remote locations, capture fast movements, and are inexpensive. However, they also have limitations such as imperfect detection, insufficient power supply, and insufficient data storage (28). Convolutional neural networks (CNNs), where filters are applied to the input data to extract features and spatial dimensions of images are reduced, are therefore increasingly used to support video footage in attempts to negate the limitations.

## Response to sound in respect of threat

In respect of sound, Morton’s motivation-structural rules present that in mammals, high frequency calls relate to fear or appeasement, whereas low frequency calls indicate aggression (29). Such rules have been proven and validated across a range of species including dogs, pigs, and elk (29, 30). These studies indicate a link between how mammals convey and perceive acoustic emotions, allowing humans to determine emotional context produced by other species. Evidence of this in other species such as birds, reptiles and amphibians are supportive, though show detection of negative emotions only, e.g., fear or aggression (31). There is debate among researchers as to whether familiarity of the listener with the subject enhances context recognition. Scheumann et al. (32) stated that humans conclusively rely on experience-induced recognition rather than acoustic clues to recognize emotional context. Alternately however, McGrath et al. (33) found that 69% of human participants in their study correctly identified whether vocalizations from chickens were in positive (reward) or negative (non-reward) contexts, not influenced by previous species association or experience. This validates further research on human perception of emotion as related to other mammals and if emotional sound recognition is intrinsic among vertebrates. Interestingly, the same study found human age to be a limiting factor in the ability to determine emotional context. Older participants were less likely to correctly identify negative sounds compared to younger participants, presumed to be attributed to hearing losses.

Biotremology is a term that refers to the practice of using organs to detect communication signals comprised of vibration. The study of this has gained popularity in recent years as vibrations can be measured through various media including soil, water, and spider webs. Vibrations occur in many situations and may be intentional signals or accidental cues not meant for interpretation; they are often used by species such as kangaroo rats, drumming on the soil surface to establish territory (30).

Communication is a vital aspect of an animal’s life, being the foundation for many relationships between and intra-species (34). However, there are many constraints for effective communication in the natural world. As the general premise of bioacoustic monitoring is it being a less invasive monitoring technique, we must consider the natural environments’ impact on both communications, and the recording techniques. Variable conditions can affect signals (4): atmospheric conditions such as wind, climate, topography and season may alter the necessary volume of animal calls to travel greater distances. In some cases, rain has been found to halt courtship proceedings; masking by natural sounds such as rivers and streams dilute animal calls, interfering with their communications. However, a phenomenon known as “the cocktail party effect” (the ability to hone in on one ‘signal’ by selectively filtering all other stimuli, i.e., focusing on one person talking in a sea of conversation) has been found to be useful to species such as frogs who can recognize single signals in a cacophony of choruses; and geographic variation has been known to overtime separate members of the same species into further populations, often promoting novel dialects alongside.

Individual species calls can be in response to many stimuli and situations such as, predatory, alarms, foraging success, and danger (29). Furthermore, the ability for animals to recognize context in other species designated sounds can supply personal gain, e.g., increased foraging success and decreased need for vigilance. Novel sounds have been known to spark wariness across species indicating that regardless of intended context, innate fear of new sounds may instinctively produce a negative reaction in the receiver (35).

## Sound and smell combine to enable communication

All living organisms use chemicals for communication, both with each other and the environment (36). These chemicals are referred to under various names throughout the scientific literature, though the most common and recently coined is “pheromone” (37). Chemical pheromones generate species-specific reactions that provoke certain behaviors, e.g., courting behaviors like lordosis, a sexually receptive posture (38).

As is often the case, the murine model system has very much enabled our understanding of the links between olfaction, urine, urinary proteins, feces and acoustic communication. Murine major urinary proteins can retain pheromones within molecular cavities, slowing release of chemical scents. Therefore, mice can identify other individuals, engage in reproduction, detect wellness/illness, and determine hierarchy. Ultrasonic vocalization then ensues based on olfactory input as related to pups’ status and communication, reproduction, and hierarchy stabilization (lower pecking order males will vocalize less). Female mice “sing” in a repetitive fashion (39) while depositing large amounts of urine. Given the immense body of research, it is no surprise that the molecular biology of vocalization has been charted to some degree (see below). In both rats and mice, emotional contagion by communicating fear to others is important. Silence is a form of communication for this purpose (in the form of ceasing rustling for communication), a communication cue that can spread across a population (40).

Furthermore, male mice have been found to release urinary pheromones that stimulate aggressive behavior in other males, detected by dissociated vomeronasal neurons (41). Chamero et al., (36) found that there are at least two populations of these neurons that can detect said pheromones, both sufficient to encourage aggressive behaviors among males. However, mice also communicate through vocalizations within the human hearing range, and ultrasonic vocalizations in social contexts including pup separation, territorial issues, and courting (42) showing the complexity of animal communication-orientated behaviors (43).

In human-audible range, pigs have vocalization of approximately 20 distinct categories associated with emotion (positive/negative) and context (feeding, nursing, distress, aggression). Their calls encode information about size, sex, and emotional state that are relevant to good husbandry and livestock management. Another reason to study these communications is the similarity between the pig and human brains; in the future it is expected that autism pig models and neural disorder pig models will be developed to study white matter pathology and social behaviors.

**Figure 2:**
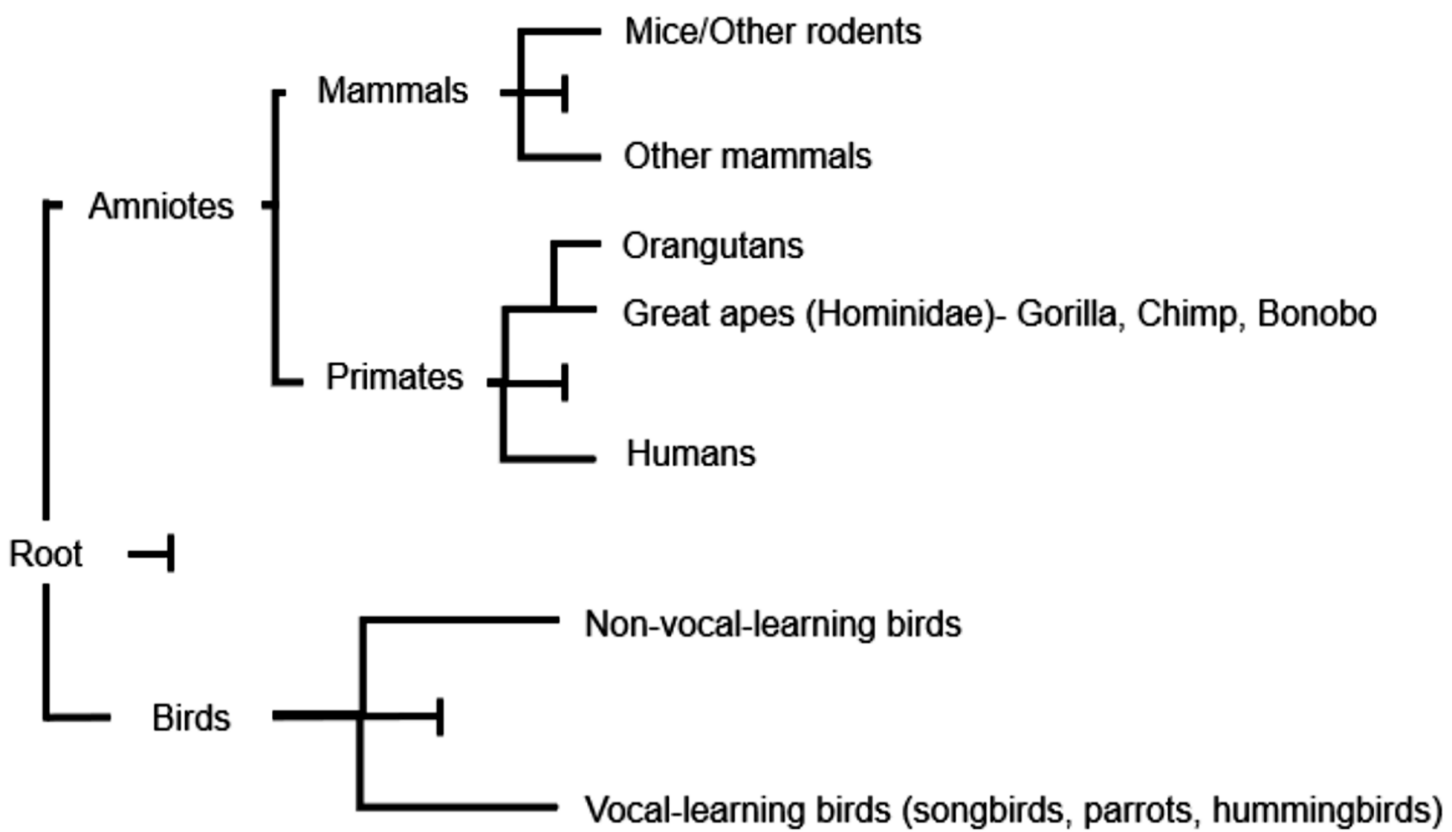
Evolutionary tree depicting the evolution of the FOXP2 gene. Two mutations in human FOXP2 have played a key role in the evolution of language. These mutations are not seen in chimpanzees and other primates.

Molecular basis for human language is now well described. FOXP2 is a transcription factor with many downstream target genes that are involved in brain development (Figure 2). This includes SRPX2, a gene involved in synapse formation. Mice do not have language, but they do use the same mechanisms as humans for (ultrasonic) bioacoustic communication (Figure 2). When the human ortholog of SRPX2 is expressed in mice they produce different syllables and call patterns in their ultrasonic communication (3). This linkage can be further demonstrated by the fact that FOXP2, the human “speech” gene, has an ortholog in mice. When one copy of the FOXP2 gene is knocked out in mice, their ultrasonic communication is compromised. This demonstrates bioacoustics is built on a foundation of shared neural architecture in mammals.

## Research on nonhuman bioacoustics in the wild

Bioacoustic listening provides the opportunity to monitor wild species and learn about their communication. In practice, this monitoring has shown differences across a variety of species. For example, some animals (e.g., whales) can alter their sound properties to account for changes and interruptions (44), whereas some species (e.g., ants) are more limited with this (45). Furthermore, some species are born instinctively knowing their species’ vocalizations, whereas others must be taught calls or songs (46). Interestingly, there is evidence of prey species coevolving with predators or similar prey species to avoid predation (e.g., some fish have evolved to hear high-frequency dolphin signals) (47). Bioacoustics can be used to monitor populations. In 2023, the Australian Wildlife Conservancy secured Government funding to implement a monitoring program for threatened species (kowari, bilby, plains-wanderer) across a 629,000-hectare cattle ranch to provide acoustic data for analysis and conservation (48). While these species have a limited vocal range they can be used to monitor population numbers and stressful events. This can also be applied to livestock (see below). In addition to these threatened species, the data also provides insights into the behavior and distribution of predators such as feral cats, foxes, and dingoes.

Research in bioacoustics has shown that sounds produced throughout the animal kingdom are indicative of territorial responses, interactions, mate impression and orientation (18). Additionally, new species discoveries can be attributed to sound analysis via decryption of distinct signals.

### Whales

Whales have cognitive abilities and societies proportional to humans, but their oceanic habitat provides a significant ecological difference (49). Whale songs are lengthy, rhythmic, and constantly evolving (50), presenting as structurally parallel to human speech in frequency, tone, and punctuation by silence (51). Additionally, they have a vocal repertoire of over 40 unique non-song social calls which vary with social complexity and size of groups (52, 53). Sperm whales have been studied in this respect and display vocalization via clicks and silences that show apparent sentence formation (syntax) and a vocabulary. As with mice it can also be said that distinct groups have dialects. Machine learning techniques have been implemented in Dominican sperm whales, demonstrating the feasibility of establishing a CNN to understand meaningful whale vocalizations (49). Recently, McCowan *et al.*, (54) successfully conducted a human-whale acoustic and behavioral interaction with a female humpback whale in Southeast Alaska. The so-called speech gene (FOXP2) in whales is closer to the non-human mammal orthologs with no whale-specific changes, thus on a molecular level, major changes have not occurred in whale evolution, yet they have one of the most complex non-human communication systems in mammals.

### Primates

All gibbon species are threatened with extinction; therefore, monitoring them for conservation purposes is of utmost importance (55). As they are primarily found in remote areas, human monitoring is costly and labor intensive. Therefore, passive acoustic monitoring is employed to monitor their presence by vocalization frequency and intensity. One study outlined the difficulties of the available wildlife monitoring equipment and investigated the use of smart phone technology to detect the presence and distribution of gibbons with successful results from more recording locations, with less effort, and fewer surveyors (56).

Two amino acid substitutions (T303N, N325S) occurred in the human FOXP2 after the divergence from the chimpanzee lineage. Chimpanzees, gorillas and orangutans have the same FOXP2 gene in terms of amino acid sequence. Neanderthals share the same sequence as seen in humans. When expressed in mice individually these two amino acid changes each altered neuronal synapse efficiency (57). Other effects of altered neurons in mice are also seen and link to speech. The key conclusion of work on this molecular evolution of the speech gene demonstrates the major leap with respect to language has arisen through FOXP2 gene enhancer structure and also the FOXP2, T303N, N325S mutations. Communication with animals does meet this biochemical/neurophysiological impasse.

### Tigers

The Prusten Project^2^ uses bioacoustics in conservation-animal behavior, and ecology studies. Using acoustic monitoring, the Project group study social vocalizations of wild tigers; the aim being to determine whether tiger’s vocalizations change depending on sex, age or individual “personality.” The project provides data used to increase protection efforts in heavily poached areas, as well as increasing understanding of how tigers communicate. The monitoring has also provided insights into how prey species alert other members of their species to the presence of tigers. Additionally, bio-acousticians have recorded and analyzed tiger roars to determine the reason behind their paralyzing effect; they concluded the likely cause being that when tigers roar, they produce loud sounds well below 18 Hz in frequency, which still produces a significant vibration to animals that cannot hear that frequency range. In summary tigers, as with many other species, have a genetically “imprinted” pattern of vocalizations with little evidence of extemporization via vocal learning.

### Elephants

Elephants do display elements of learning with respect to their vocalization patterns and adjust sound production depending on vocal context and in response to specific cues or auditory inputs. It is argued that elephants have brain structural features like songbirds and humans that enable this vocal learner capability. Elephants produce low frequency sounds. Recently, it has been discovered that elephants address each other using name-like calls, individual to each member of the herd (58). This differs from many other species who simply imitate the receiver’s call. Elephants are one of only a few species to date that have exhibited vocal learning, i.e., are able to mimic new sounds, though the application for this learning remains unclear (59). However, their FOXP2 gene has not acquired mutations akin to those seen in humans and neanderthals associated with advanced speech.

‘Bee fences’ are used as a deterrent in Africa to ward elephants off from raiding village crops (60). It has been documented that elephants fear bees and run away from their buzzing sounds, also producing a low grumble that warns the rest of the herd of bee presence. As other forms of fencing are either too expensive, dangerous, or unsuccessful, beehives are set up along the border of the farms. If disturbed, the bees will react and deter the elephants. Furthermore, motion sensor speakers have been successfully trialed that mimic the bee’s buzzing noise when an elephant approaches to ward them away from the area. Thus, this creates value in bioacoustics for harmonious human/elephant cohabitation.

### Fish

Fish can produce sound in a number of ways (e.g., vibrating the swim bladder). These acoustic signals can be used to indicate stress, mark territory, or in sexual reproduction associated activities. Distinguishable sounds are produced by fish during the mating season, whereby bioacousticians can analyze behavior patterns. Body size, stamina, and readiness to mate can all be signaled via sound. These sounds are low in frequency and intensity, only spanning large distances if in mass groups. Additionally, fish species have been found to produce distinctive spawning sounds leading to the development of a ‘spawn-o-meter’- a passive acoustic technology that documents when and where fish spawn by counting specific sounds over a period in a single site (61). A few fish species show evidence of limited vocal learning (e.g., damselfish), but these are exceptions.

### Reptiles and amphibians

Crocodiles, geckos, frogs, and toads have been monitored for their vocal repertoire (62, 63), though sound repositories for these species are limited and there has been a lack of analysis (64). Notably, signals change in exotherms with ambient temperature (65), which has been used to differentiate between populations of the same species in different climates.

### Bats

Bats are well known for their ultrasonic communications termed ‘echolocation’. The study of these sounds allows for data collection relating to bat distribution, behaviors, and species identification. Monitoring of bat dynamics shows them as potential bioindicators, denoting ecosystem health, due to their sensitivity to habitat conversion and climate change (66). Almost 80% of bat species use echolocation to search for prey, dodge obstacles, and communicate (67), controlling and improving their sensory location with their mouth gape (68). Bioacoustics offer a non-invasive way to study these interactions and monitor populations. Furthermore, concerns that bats are involved in the spread of viruses such as COVID-19 highlights a need to also track and monitor their interactions with humans. Artificial Intelligence tools have been proposed as a way to do so (69). Deep learning algorithm software such as BatDetect and Waveman have been developed to increase detection performance of echolocation calls in bats (66, 70). Annotated datasets of bat vocalization related to context and age exist and enable further understanding of communication to be developed (71) and there is clear evidence of vocal learning in younger bats (20). Vocalizations carry, at a minimum, information about the “speaker,” call context, behavioral response, and the vocative case (addressee) (72). In fruit-bats, their vocalization can convey contextual information via several sounds, though the order of the sounds within the sequence is unimportant for context (73). Importantly this work used neural network-based research to identify these phenomena, showing the future direction of this research for veterinary medicine in companion animals and livestock. Echolocating bats have seen accelerated evolution in FOXP2, with many amino-acid changes across species, far more than most mammals, but these changes are not the same as the two key human/neanderthal mutations. The bat-specific mutations are thought to control echolocation and vocal learning. This demonstrates the critical role FOXP2 has in neuroregulation and bioacoustics adaptation; it demonstrates basic molecular biology is changed by mutation, but human/neanderthal communication was and is at a different level via complex mechanistic changes underpinned by 2 mutations. It is unlikely we can have a conversation with a bat in respect of an ideas exchange.

### Birds

Most bird species do have the ability to learn vocalizations. Their range and type of calls and their understanding thereof is embedded in the genome or epigenome of the species. There are exceptions. Vocal-learning birds learn through tutoring, imitation and repeated practice. Their neurological structures are different to non-vocal learners. This group of birds includes parrots, hummingbirds, and songbirds. FOXP2 has a characteristic expression pattern in a brain structure uniquely associated with learned vocal communication. The striatal nucleus Area X, which is key for vocal learning shows differential expression of this key “talking” gene between vocal learners and non-learners (74).

In wild birds there is a high song variability between individuals, which can make species identification difficult for both humans and technology (75). However, flight calls can be monitored to identify flight patterns and migration. Acoustic monitoring has also displayed evidence that between bird species, some females prefer highly stereotyped courting songs, whereas others prefer males with an ability to improvise (10). See (76) for recent work using artificial intelligence to decipher features of birdsong. We will consider poultry as a separate case, below.

## Research on nonhuman bioacoustics in domesticated animals

### Dogs

Much of the literature pertaining to bioacoustics in dogs relates to the human-animal bond and how humans analyze canine vocalizations for our benefit. Research shows that humans can recognize the context of dog barks (e.g., play or threat) independent of previous experience with dogs (77). Unlike cats, and due to their social relationships, the vocalizations between dogs are the same ones used from dog to humans (78). In terms of dog-dog communications, studies have shown that dogs are able to distinguish between ‘familiar’ and ‘stranger’ barks via context, with it also being evidenced that dogs extract detail from barking (79). Generally, vocalizations in dogs can be categorized into bark, howl, growl, whine, yelp, snore, groan, and grunt, all expressing different contexts (78). However, the dog soundscape remains largely unknown. Savel and Legou (80) have recently published a study which proposed a model of the soundscape, listed acknowledged sounds, and identified the potential relevance between environmental sounds and behaviors. One study investigated whether machine learning-based acoustic analysis (“EmoDog” (Emotional Dog Corpus) dataset- 226 bark sequences from the Mudi dog) can recognize context and emotion in dog barks, with successful results; the feature sets have been found to be able to classify context, perceived emotion, and intensity of dog barks (81). The study also concluded that due to similarities in emotional responses and vocalization anatomy, changes in emotion produce similar effects independent of mammalian species. Another study found that sex and age of domestic dogs can be estimated using sound analysis (82); also providing indirect proof that barking is an important component of information transfer between dogs.

The human animal bond involving dogs is special in the sense that dogs are sensitive to nuances of human speech and can detect emotional context and phonemes/phonetic differences. In respect of their vocalization, dogs use a wider range of tones than other animals to communicate with humans and this aligns with their ability to display context in their vocalization (stress, pain happiness). Wolves do not have these capabilities and unlike domesticated dogs do not tend to make eye contact with humans. Dogs bark more often than wolves and wolves have a far lower vocal range than the domesticated dog. These more varied utterances are also directly targeted at humans which is uncommon in other species. Critically important given the adaptation of dogs to humans over the past few thousand years, is the fact that they can distinguish meaningful words from humans, which, for example, a chimpanzee or orangutan cannot do.

Playing music for puppies/dogs has been shown to reduce anxiety; more specifically, classical music due to its slow tempo and low frequencies (83). As such, various CDs and playlists are marketed to play when dogs are left home alone. Also, radio sounds and noises have been proven useful for acclimatizing young puppies to environmental sounds (84). This is believed to be due to a perceived safe environment and the mimicking of maternal heartbeat patterns or breathing of close relatives.

### Cats

Cats as compared to dogs are weakly responsive to human language, have not learned to detect context specific human language, and do not display the slightly enhanced bioacoustics learning that dogs have. Nonetheless, cats have been studied intensively relating to bioacoustic monitoring due to their unique ability to purr, found more recently to occur both actively and passively (85). In this context domesticated cats do show increased vocal plasticity compared to wild cats and display increased responsiveness to cues generated by humans and a greater tolerance for general human-generated sounds. There is no evidence of altered vocalization structures between domesticated and wild cats.

Nonetheless, cats are said to use vocal communication during three interactions, parent to young, agonistic, and mating; though it has since been found that they also use specific vocalizations during socialization (86). “Meowing” is a mainly human-directed vocalization that cats use to communicate their emotional state to their owners: Prato-Previde *et al*. (87) found that accuracy of human interpretations largely rely on previous experience with cats and level of empathy. Purring and meowing do relate to emotional state though further work is required in this area (88), potentially using data from wearable devices. Raccagni and Ntalampiras (89) investigated whether machine learning based on the public audio dataset “CatMeows”^3^ could be used to differentiate and identify cat breed with successful results.

Like dogs, cats have also been studied for their reaction to music. Specifically, biometric data, collected by smart collar sensors, has been used to produce “science-based music” designed to have a calming effect on cats (90). One of the more interesting ideas to use this human animal bond for product development is to use artificial intelligence and machine learning to modulate companion animal behavior with sound. For example, to regulate whining, barking, boredom, agitation and generate wellbeing.

## Livestock

Humans are dependent on plants and animals as a food source. Therefore, as populations increase, demand for food increases, meaning we are continuously researching methods to improve production. The welfare of production animals is an important aspect of rearing, as it improves the quality of the carcase (91). Bioacoustics can be used to monitor an animal’s weight, behavior, and sounds to determine the welfare status and allow for human interference if necessary. Using software to monitor these aspects in farm animals has been shown to be more efficient in detecting issues than stockperson judgment (92). The detection of diseases can help to prevent spread to both humans and other animals. In turn, this can reduce the economic and mental health impacts of disease on farms.

**Figure 3:**
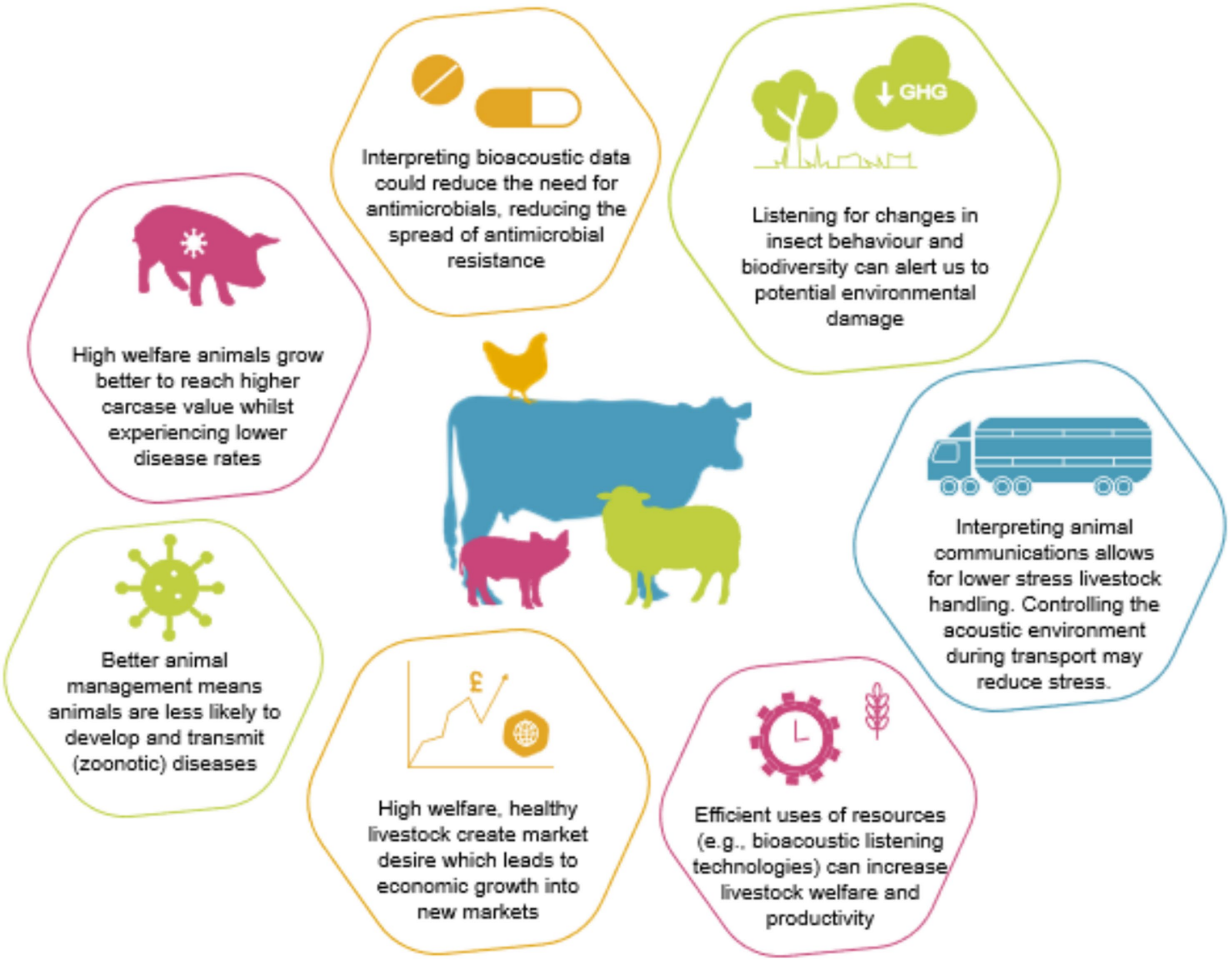
A diagram to depict the benefits that bioacoustic listening and analysis may have for rearing production animals. Examples of the value of new knowledge is given. Transportation of animals, for example, results in high mortality and morbidity rates and any communication platform that could calm livestock before or during travel would improve animal wellbeing.

Bioacoustic analysis can be used to assess health, physiology, behavior, and affective state of an animal (93). The key issue with livestock, is how do we ethically and meaningfully use animal language to improve the life experience for the animal? Figure 3 outlines the secondary benefits that may ensue, including better health, welfare, and economic enhancement for livestock owners.

Non-human vocalizations are generally placed in two categories: calls, and songs. Calls are shorter and simpler, only composed of one or two sound units, whereas songs are longer and more complex, composed of several sound units (93). Songs are used in only a few contexts including territorial defence and mate attraction whereas calls may be used more widely for example during alarms or contact (94). Livestock calls are a matter of much interest as they offer opportunity to improve welfare.

Distress calls from newborns have an innately powerful effect on the maternal nature in many species, to promote reaction and infant survival (95). Acoustic stimuli are critical throughout the growth of mother-newborn bond, in sheep especially, with ewes able to identify their young as early as 6 hours post-partum (increasingly so with experience) (96). Cattle have also been found to vocalize more during the first 24 h of calf birth, to teach the calf their call; and when separated from their young (97). Similarly, piglets have been found to respond more strongly to barks of adult sows than their peer piglets (98). With an increased demand for meat and dairy produce, farming practices constantly being in the public eye, and a shortage of skilled workers, the use of technology for livestock rearing and monitoring provides opportunities for objective, cost effective, and non-invasive methods to improve welfare and efficiency (97). As public concern for farm animal welfare increases, we must consider alternative methods to decode their emotional state (99), limited as it may be. There is no available open-source dataset for farm animal welfare use of which we are aware, therefore a huge step forward would be the creation of such (5). Providing animal behavior and welfare scientists with access to a database to input information surrounding recorded vocalizations including animal age, size, weight, and context and location of sounds, would provide large data-sets currently not available elsewhere, giving opportunity for data to be analyzed against the five freedoms (Table 1) to determine an animal’s quality of life using bioacoustic methods. Again, it is important to stress that vocalized exchange of information with livestock by a human is unlikely due to the (molecular biology-based uses described).

Vocalizations of farm animals can give us insights into their welfare state. For example, analysis of distressed utterances within an abattoir may be used to improve the experience for the animals (100) and could be ameliorated with positive sounds. The use of acoustic monitoring could also be integrated into precision livestock farming, such that detection of vocalizations may indicate a need for environmental changes that can be programmed to occur via computation-based learning (5). For example, detection of animal calls that indicate low airflow tells the computer-based system to open windows for increased ventilation.

Cows are able to hear and act on simple cues from humans (move, come) and can respond differently depending on emotional tone, pitch and volume. In this respect cows can be trained and their mood altered by humans. Like cats or dogs; heartbeat, music, and other sounds may improve herd management.

### Ruminants

Ruminant ingestive behavior has been successfully evaluated using bioacoustics; this is an attempt to automate a manually dominated diagnostic method (101). For example, machine learning has been applied in sheep to identify and classify foraging behaviors; the aim of this being to promote practical applications of ruminant foraging behavior monitoring (102). Additionally, ewes and their lambs recognize each other by their calls (103). However, it has been discovered that the frequency of calls differs depending on number of offspring, with single lambers producing lower mean frequencies than twin lambers; the same findings were apparent with lambs, in that those without siblings produced lower mean frequencies than those with (104).

A difficulty presents itself when lending bioacoustic monitoring to welfare indicators in sheep as there remains gaps in the research to determine whether vocalization parameters indicate positive or negative emotions in sheep (99). For example, teeth grinding is a major indicator of pain and discomfort in sheep (105); this can be monitored, though it would need to be differentiated from cudding sounds. Higher standing and walking behaviors are also associated with stress and/or fear in sheep; Papadaki et al. (104) found that more movement correlated with increased frequency of vocalizations suggesting that greater vocalization frequencies are stress related. Additionally, they found that vocalizations differ between species of sheep, also found during another study which demonstrated that voice analysis of sheep can be used to estimate genetic distance (106).

Cattle vocalizations have been shown to provide information about age, gender, breeding status, pain, separation, hunger, and thirst (97, 107). Jung et al. (108) developed a CNN model for classifying cattle voices with 94.18% accuracy, and 81.96% accuracy in monitoring cow status. Additionally, cattle calls can be used to identify individuals in a herd due to the inter-cow variability in vocal characteristics, paving the way for identification of individual wellbeing status (97). Acoustic monitoring has been used to determine the relationship between grazing behaviors and dry matter intake via analyzing number of bites it takes for the cow to finish a mouthful (109).

### Poultry

It is important in terms of livestock husbandry to understand that chickens are not capable of vocal learning (110). Vocal learning is a trait found only in a few bird types such as songbirds, parrots, and hummingbirds. Poultry have an array of different calls, but they are part of a genomic or epigenomic preprogramme that does not contain vocalization learning.

Chickens nonetheless are highly vocal animals, thus excellent models for acoustic monitoring methods. Despite morphological differences, male and female chickens have similar acoustic parameters, differing only in frequencies (111). Adult males have developed different calls to distinguish between land and airborne predatory threats (5). Moreover, distress calls in chickens have been suggested to distinguish welfare status, termed in literature as ‘iceberg indicators’ (112). Healthy and non-healthy chickens have been successfully identified from their sound signals using a voice activity detection (VAD) algorithm (113). Additionally, stress levels of young and adult chickens have been successfully monitored using acoustic parameters of vocalizations (114, 115). Further work is required to predict illness and potentially help with diagnosis as well as identify stress early in the event and signal this to the stock handler.

### Pigs

Pig vocalizations are three-fold, squeals, grunts, and barks; each identifying a different emotional state, found to relate directly to body size. They can produce over 22 different types of vocalizations, recognize human voices and tone content (e.g., harshness), have a well-developed auditory complex in the brain, and are very capable, relatively, in socialization via acoustics. Pigs kept in isolation produce significantly fewer vocalizations overall than those in enriched social situations (5). One study investigated whether there is an acoustic difference between piglets that are thriving and not thriving following weaning, indicating that those failing to thrive produced longer, higher-pitched calls (116). Additionally, it has been suggested that pig vocalizations carry pain information, therefore providing an appropriate method for measuring welfare (e.g., presence and prevalence of tail biting) (5).

Crushing of piglets is a significant loss to the pig industry (100). Therefore, given that piglets produce identifiable vocalizations during this time, bioacoustic monitoring in farrowing scenarios could highlight these instances, promoting intervention or initiating system changes. Schön et al. (117) used linear prediction coding to identify individual piglets through vocalizations and classify calls into stress and non-stress related. Using vocalizations to determine stress levels has since been further confirmed (118).

Bioacoustic microphone positioning has been used to localize coughing sounds within pig pens to identify diseased individuals, provide opportunities for early treatment, and reduce the spread of respiratory pathogens (119).

## Environmental management

### Insects

Insects play a wide array of ecological roles from ecosystem balancers to disease vectors. As they are heavily affected by climate change, there is an ever growing need to monitor their movements, distributions, and behaviors to subsequently and indirectly monitor the global climate. A promising method for this is bioacoustic monitoring (120).

Insects produce loud audible sounds over a range of frequencies, including communication via vibrations (121). The use of sound recording in insects provides the potential for speciation during mate finding. Song structures that are only differentiable by sound recording can help to describe and identify new species, as well as recognizing known species (122). In 2007, analysis of sound recordings led to the discovery of novel information regarding *Cicadetta* biogeography (123). Parasitoid insects have also been found to use sounds to locate their host; insects such as cicadas and crickets ‘sing’ to each other, flies use these sounds to locate them and lay eggs on or near the host that hatch into larvae, burrowing into the host and consuming them from the inside out (124).

Insects communicate via six pathways: hormones, sound, pheromones, motion, exocrine glands, and enzymes (37). Therefore, manipulation of sex pheromones in insects has been highlighted as an alternative method to pesticides (37). The idea behind this is that the insects are more focused on chasing the source of the pheromones that indicate mating opportunities, than finding and damaging crops. As pheromones are such volatile compounds, the main challenge is to sustain a regulated release over a longer period in the necessary volume to attract the insects. The idea works by disrupting natural mating practices in the insects. Some males searching for mating females have been thought to expend their mating energies pursuing the artificial sources instead, and it is thought that constant exposure to low levels of the artificial pheromone diminishes the ‘navigation system’ of the insects by affecting their antennal receptor sites.

### Climate change and the use of bioacoustics monitoring

Climate change is a major threat to many plant and animal species; therefore, bioacoustic monitoring allows use of sound data for conservation efforts, e.g., monitoring coral reef activity for changes in sounds. The increase of man-made noises in the oceans has been found to interfere with marine life and behavior, particularly by masking animal communications (61). For example, marine larvae have been found to critically rely on acoustic cues to orient to an appropriate habitat, therefore, alleviation of noise pollution in the ocean is more important than first thought (125, 126). Furthermore, there have been cases made for quieter diving technology as the associated bubble noise disrupts not only recordings, but animal behavior under the water, leading to disturbances in courtship behavior and spawning sounds in observed species (127).

Similarly, monitoring bioacoustic signals in the wild can provide information regarding movements and pollution. Combinations of bioacoustic tools and monitoring systems with analytical technologies allows us to analyze changes in distribution and behaviors, and effects of human activities (128). Unfortunately, humans produce many significant air-borne sounds that overlap with animal hearing frequencies such as road traffic, wind turbines, airports, urban environments, and construction (129). As many animals, in particular insects, have such sensitive hearing, these noises are highly likely to disrupt their communication methods. Dealing with such noise levels can affect many species not only behaviorally, but physiologically. In some cases, noise levels may be high enough to cause hearing loss, reduced foraging, and a stress response (130, 131).

Recent research has discovered that plants also produce sounds in accordance with their needs, more specifically emitting ultrasound waves when stressed (129). The he frequency at which plants make their sounds is within range of some animal species (20–100 Hz). For example, it is hypothesized that moths may use the tree soundscape to identify an appropriate branch to lay their eggs; a ‘stressed’ tree emitting such ultrasound waves may be more fragile, and hence riskier, for egg survival.

During the COVID-19 pandemic lockdowns, anthropogenic noise levels were significantly reduced globally (132). For some species, a reverse Lombard effect (an involuntary tendency to increase vocal effort when surrounded by loud noise) was noted, meaning that due to the reduced surrounding noise levels, some bird species were observed to lower their vocal efforts accordingly. This highlights the negative effect that anthropogenic noise can have on animal communications.

## Discussion

As stated, acoustic cues are used during communication. By monitoring these cues, we not only learn about individual species variation, but provide a baseline to measure the extent to which an increase in anthropogenic sounds may negatively affects these communications. In turn, this can lead to insights into how such interruptions to the surrounding soundscapes may affect the overall health and welfare of many species or ecosystems. Anthropogenic noise pollution has been reported to affect sexual and social signals in fish, amphibians, mammals, and birds, having a direct impact on breeding, socialization and population growth (133). However, it has been noted that overtime, fish can habituate to some noises (127). Additionally, humpback whales have been found to have changed their vocalizations due to interference from shipping noises (134). Some species have even adapted to use the noises from ships to their advantage, indicating where potential feeding grounds lie (34).

Humans have domesticated many species from companion animals, to farmed, to zoo. It is therefore important that we make efforts to understand their vocalizations and behaviors to ensure, not only the welfare of the animals, but the welfare of the keepers. The ability to understand vocal cues related to stress, pain, and fear in other species can provide keepers with important information on how to approach and work with the animals calmy, safely and effectively while also enriching their environment (135). Additionally, attributing vocal cues to emotions increases the likelihood of keepers anthropomorphising their animals, likely in turn increasing animal welfare.

Furthermore, not only can bioacoustic monitoring provide insights into the communication between non-human species, but it may also directly translate back to human socialization. Analysis of vocalizations in primates is used as a model to monitor vocal development in autistic children; vocal interactions can be used to monitor sociability and ability to learn societal rules (136).

In respect of the molecular machinery of bioacoustics and communication, the FOXP2 gene is not a master regulator of language but it is critically important in bioacoustics in mammals and has been used here to stress that biochemistry underlies bioacoustics beyond sound generation. Two key mutations in FOXP2 in humans plus altered expression patterns (in part due to enhancer region mutations) have in part defined the difference between humankind and other mammals in respect of language and communication. Given the clarity of the effects of FOXP2 on neuronal development and the fact the human ortholog has major differences in its actions and downstream targets, the idea that a “Dr Doolittle”-type engagement between other species and humans remains remote on molecular, physiological grounds. Nonetheless, pigs, cows, dogs, and other species can engage with humans. It is to be hoped that we can use this two-way communication to improve animal health and welfare.

While bioacoustic monitoring has many existing applications worldwide, there remain areas for improvement. AI should be used only as an assistant to expertise, not a replacement. Alternative programming of learning models creates differences in knowledge and analytical abilities of the software. A review of deep learning models found that no single model has been found to significantly outperform others across all features, concluding however, that model performance can vary significantly (137). One such way to increase the opportunities for researchers alike, is increasing the frequency and performance of openly available data (138), as only 21% of published papers share their recordings for others to use (139). Most proteomics/metabolomics/transcriptomics and DNA databases are huge but are published alongside the peer reviewed journal content to ensure maximum use of data.

A significant pitfall to analyzing bioacoustic data is that deep learning techniques carry a high bias risk; therefore, the development of reliable systems would lead to equal representation of analytics. Similarly, there is somewhat of a species bias throughout bioacoustics research, with under-studied species needing to be recognized more (140). There is also debate about the best user interface for deep learning, as many produce outputs as Python scripts that may be inaccessible to broader communities of interested parties; however, Stowell (138) states that it is likely that algorithms will be available as installable packages in the future and artificial intelligence approaches are now writing code to specified requirements.

While progress in the advancement and understanding of bioacoustics research is exciting, the ultimate value depends on realistic opportunities for integration into routine veterinary practice. Data interpretability, educational training, and usability are all factors in adoption of bioacoustics techniques by farmers and vets alike. These are coupled with issues of initial hardware and service contract costs for the purchaser. Artificial intelligence-based solutions to data interpretation will drive down costs as will the ever-decreasing cost of computer silicon chips for hardware. These real-world issues will drive customer views on return on investment in bioacoustics solutions.

## Conclusion

This review outlines that there are numerous available and ongoing studies regarding the applications, techniques, and methods of bioacoustic monitoring for animal vocalizations as indicators of distribution, interactions, and emotional state. By understanding the vocalizations, we can understand what they represent and respond effectively. These signals provide information not only about the animals, but about their environment, climate, and behavior. Analysis of these components combined, provides decoded insights into physiological, emotional, and behavioral aspects of both groups of, and individual animals. Through existing monitoring systems, we know that many species have altered their own vocalizations to communicate with other species within their environment, in attempts to cohabit effectively. We simply must do the same.

## Footnotes

1
https://www.openacousticdevices.info/live

2
http://www.theprustenproject.org

3
https://zenodo.org/records/4008297
